# Bats in a Farming Landscape Benefit from Linear Remnants and Unimproved Pastures

**DOI:** 10.1371/journal.pone.0048201

**Published:** 2012-11-14

**Authors:** Pia E. Lentini, Philip Gibbons, Joern Fischer, Brad Law, Jan Hanspach, Tara G. Martin

**Affiliations:** 1 The Fenner School of Environment and Society, The Australian National University, Canberra ACT, Australia; 2 School of Botany, The University of Melbourne, Parkville VIC, Australia; 3 Faculty of Sustainability, Leuphana University Lueneburg, Lueneburg, Germany; 4 Forest Science Centre, NSW Primary Industries, Beecroft NSW, Australia; 5 CSIRO Ecosystem Sciences, EcoSciences Precinct, Dutton Park QLD, Australia; University of Kent, United Kingdom

## Abstract

Schemes designed to make farming landscapes less hostile to wildlife have been questioned because target taxa do not always respond in the expected manner. Microbats are often overlooked in this process, yet persist in agricultural landscapes and exert top-down control of crop pests. We investigated the relationship between microbats and measures commonly incorporated into agri-environment schemes, to derive management recommendations for their ongoing conservation. We used acoustic detectors to quantify bat species richness, activity, and feeding in 32 linear remnants and adjacent fields across an agricultural region of New South Wales, Australia. Nocturnal arthropods were simultaneously trapped using black-light traps. We recorded 91,969 bat calls, 17,277 of which could be attributed to one of the 13 taxa recorded, and 491 calls contained feeding buzzes. The linear remnants supported higher bat activity than the fields, but species richness and feeding activity did not significantly differ. We trapped a mean 87.6 g (±17.6 g SE) of arthropods per night, but found no differences in biomass between land uses. Wider linear remnants with intact native vegetation supported more bat species, as did those adjacent to unsealed, as opposed to sealed roads. Fields of unimproved native pastures, with more retained scattered trees and associated hollows and logs, supported the greatest bat species richness and activity. We conclude that the juxtaposition of linear remnants of intact vegetation and scattered trees in fields, coupled with less-intensive land uses such as unimproved pastures will benefit bat communities in agricultural landscapes, and should be incorporated into agri-environment schemes. In contrast, sealed roads may act as a deterrent. The “wildlife friendly farming” vs “land sparing” debate has so far primarily focussed on birds, but here we have found evidence that the integration of both approaches could particularly benefit bats.

## Introduction

Agricultural intensification and associated habitat fragmentation are key threatening processes for wildlife [1]. To mitigate negative effects associated with these, Agri-Environment Schemes (AES) have been established in many regions of the world, which offer farmers financial incentives to plant and protect vegetation, use fewer agrochemicals, or employ alternative grazing regimes [2]. Because all species do not necessarily benefit from such ‘wildlife-friendly farming’ measures [3], [4] some propose that investments could be better spent establishing separate conservation reserves, an approach known as ‘land-sparing’ [5], [6]. However, most existing work has focused on birds, and other, more cryptic groups may respond differently to AES. For example, microbats are highly mobile, are able to exploit patchily-distributed resources and retained features in the landscape, and often constitute a large component of the mammalian fauna in agricultural environments [7]. They also exert top-down natural control of arthropod pests that have considerable impacts on crop yield [8], [9]. Depending on reproductive condition, a single microbat consumes 40–100% of its own body weight in insects per night [10].

To date, there is a lack of consensus as to how to best manage for bats in agricultural environments. In Europe, AES which are primarily designed to support birds, invertebrates, and plants [2] bring varied benefits for microbats. For example, Wickramasinghe et al. [11] recorded higher levels of bat activity and feeding on organic compared to conventional farms, whereas Fuentes-Montemayor et al. [12] concluded that AES-participating farms supported lower activity of two *Pipistrellus* species and their invertebrate prey. These conflicting results may be partly attributed to the fact that bats often use complementary habitats to fulfil life-history requirements. Whereas undisturbed remnants with many old, hollow-bearing trees are favoured for roosting [7], foraging activity is often higher near trees in open areas and along edges [13], [14] because vegetation clutter can inhibit flight for some species [15]. ‘Roosting’ and ‘foraging’ habitats can therefore be quite different and located several kilometres apart [7] despite potential energetic costs of commuting [16], [17]. Several AES target linear features such as hedgerows and field margins, which are analogous to other features that transect agricultural landscapes around the world, including living fences [18], treelines [19], and road reserves [20]. Managed well, such linear features may reduce the energetic cost of commuting for bats by providing suitable roosts close to open foraging sites, or by functioning as corridors for movement [21], [22].

An Australian example of linear features are ‘stock routes’, which form a network of roadside corridors of remnant vegetation. These were originally established for the transport of livestock ‘on the hoof’, and were placed in low-lying, fertile portions of the landscape close to freshwater [23]. Because bats generally prefer to forage and roost over fertile geologies or in close proximity to water [24], [25], and many stock routes support old trees [26], they should constitute valuable bat habitat. Stock routes (“linear remnants” hereafter) also vary greatly in width, vegetation condition, and intensity of surrounding land use, and therefore provide an excellent opportunity to explore the kinds of management measures that should be implemented in agricultural landscapes for bat conservation. We aimed to establish (1) how linear remnants and surrounding fields differed in habitat value for bats; (2) what kinds of linear remnants were most important for bat conservation; and (3) what kinds of ‘wildlife friendly’ measures made fields better habitat for bats.

## Materials and Methods

### Study Region and Design

**Figure 1:**
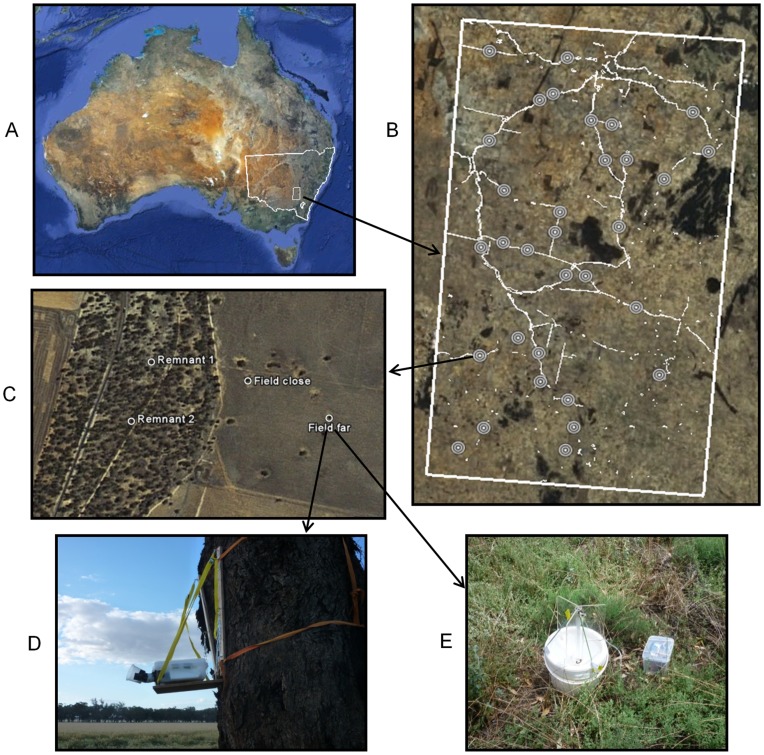
Study design. a) the study area within the state of New South Wales, Australia b) the position of the 32 study sites within the study area, with the linear remnant network shown in white c) an example of the layout of four survey points nested within a study site d) a bat detector, in a weatherproof box and with microphone funnel attached, on a platform strapped to a survey tree, and e) a black-light trap on the ground.

We studied a 15,000 km^2^ area of the “wheat-sheep belt” of New South Wales, Australia (Fig. 1a). Land use is dominated by dry cereal cultivation, as well as native and improved pastures for livestock. Prior to European settlement the area was covered predominantly by *Eucalyptus* woodlands, but it is now 84% cleared. Formal conservation reserves cover only 1.3% of the area and occur mostly on ridgelines and unproductive areas [27]. Other remnant vegetation occurs as small patches or individual scattered trees in fields, or in the public land system as linear remnants.

Our study design incorporated 32 sites (Fig. 1b); nested within each were two survey points in a linear remnant, and two in an adjacent field (totalling 128 surveys points). The two remnant survey points (‘Remnant 1’ and ‘Remnant 2’) were spaced at least 100 m apart, and the two field survey points were spaced approximately 100 m (‘close’) and 400 m (‘far’) from the remnant (Fig. 1c). Remnants ranged from narrow (38 m) to wide (570 m), and the condition of the vegetation within them from ‘intact’ (little evidence of anthropogenic disturbance) to ‘degraded’ (evidence of considerable grazing pressure or clearing). Four of the 32 remnants in this study could be classified as ‘riparian’, in they had a small stream or creek (∼2 m wide) running through them (see Appendix S1). Fields represented locally common land-uses; 12 cereal fields (wheat, barley or oats), 11 improved pastures (exotic annual grasses or lucerne/clover), five unimproved native pastures (largely perennial species), and four fields of canola (*Brassica* sp.). Access to the privately-owned fields was granted by all landholders prior to the surveys. All remnants and fields contained at least two large trees (see below). Although the region had been in drought in previous years, rainfall was higher than average in 2010–2011, restricting access to some sites. Therefore, we collected data from 114 of the 128 points only (59 remnants and 54 fields).

### Surveys

#### Bats

Bat surveys were conducted with approval of the animal experimentation ethics committee of The Australian National University, protocol no. F.ES.06.10. Microbat data were collected twice in summer 2010–2011: (1) the “maternal survey period” from 22 Nov to 22 Dec 2010, when female bats usually have dependent young, and (2) the “juvenile survey period” from 21 Jan to 14 Feb 2011, when the young had become volant. We used Anabat ultrasonic detectors (models SD I, and SD II with ZCAIM storage units, Titley Electronics, Ballina) to conduct acoustic surveys. Detectors were calibrated following Larson and Hayes [28], and set in weatherproof boxes with a cut-out for a microphone funnel. The boxes were then placed on wooden platforms strapped to trees approximately 2 m above ground (Fig. 1d). We surveyed four sites at a time, and in each placed one detector at a remnant survey point and one at a field point for two consecutive nights (total eight detectors per night). In this way, at each site two of the points were surveyed for two nights in the maternal survey period, and the other two points were surveyed for two nights in the juvenile survey period. Detectors were set to turn on at least one hour before sunset (1800 hrs), and off again one hour after sunrise (0700 hrs).

#### Arthropods

We collected flying nocturnal arthropods at each survey point using 12 volt, 8 watt black-light (ultraviolet) traps (Australian Entomological Supplies Pty. Ltd., Coorabell, Fig. 1e). Because these traps may deter *Nyctophilus* species [29], they were set out for only one of the two consecutive detector nights. We did not sample fields when livestock were present to prevent damage to equipment. Traps were placed approximately 10 m from each survey point on the opposite side of the tree to the detector, and were fitted with light/dark relay switches (Ozitronics, Melbourne) so they would switch on at dusk, and off again at dawn. Arthropod samples were stored in methylated spirits, and following the field season, were oven-dried at 60°C until desiccated. Dried samples were weighed using a laboratory balance to an accuracy of 0.001 g, and this figure was recorded as the “dry biomass”.

#### Habitat

Vegetation surveys were conducted within a one-hectare circle at each of the survey points during the two-day detector period. For all trees within this circle, we recorded the species, the diameter at breast height (DBH), presence of hollows, bark type, and stage of senescence (the last two measures were recorded only for *Eucalyptus* species; following Rayner [30]; Table S1 and Fig. S1). The number of *Eucalyptus* seedlings <130 cm tall was recorded to quantify tree regeneration, an important component of landscape function [31]. Where tree cover was extremely dense, the area around the survey point was reduced to 0.283 ha (30 m radius) or 0.126 ha (20 m radius), and these estimates were later scaled up to represent one hectare. We visually estimated the percent cover of shrub species, and measured the length and diameter of every log (fallen timber >1 m long and ≥10 cm in diameter). Two 50 m point-intercept transects were run from either side of the base of the survey tree, and at every metre we recorded the nature of the ground cover (native vegetation, non-native vegetation, rock, bare ground, leaf litter, water, cryptograms, cow dung). Finally, we recorded the type of road running adjacent to each of the linear remnants (multi-lane major highway, single-lane sealed road, or unsealed but graded laneway).

**Figure 2:**
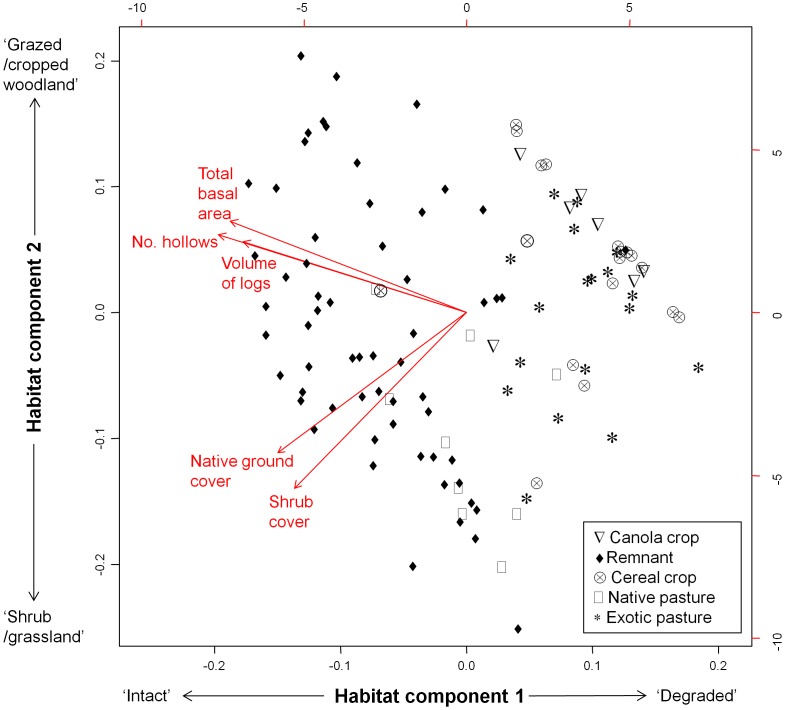
Mean dry biomass of arthropod samples collected per night in each of the land-use classes. Error bars represent 95% confidence intervals, and sample sizes are listed below each of the plotting points.

Because we would be limited in the number of explanatory variables we could include in our analysis, we carried out principle components analysis (PCA) on five of these habitat measures (total basal area of trees, number of trees with hollows, volume of logs, percent ground cover that was native, and percent cover of shrubs, see Appendix S3 and Fig. S2). This resulted in the creation of two components, which, these explained 60% of variance in habitat data (Fig. 2; Table S2). Habitat component 1 ranged from ‘Intact’ at the negative end of the scale (more trees, hollows, logs, shrubs, and native ground cover) to ‘Degraded’ at the positive end of the scale (low values for these variables). For component 2, sites that structurally resembled ‘shrub/grassland’ (more native ground cover and shrubs) scored negatively, whereas those that resembled ‘grazed/cropped woodland’ (more trees, hollows and logs) scored positively.

#### Weather

At each site, rain gauges (Nylex Rain gauge 500, Pakenham) were inserted into the ground at the fenceline in between the remnant and field survey points, and were checked daily to estimate overnight rainfall. An anemometer (Vortex Hand-Held Anemometer Pro-1200, Inspeed, Sudbury) was taped to the top of the fence to record the maximum overnight wind speeds and we also checked these daily. Finally, we used iButton thermochron loggers (model no. DS1921G, Maxim, Sunnyvale) to record ambient temperature at each site. These were set to log readings every five minutes, were tied into the finger of a latex glove for weatherproofing, then also taped to the top of the fence. Data was downloaded off the logger once a week.

### Bat Call Analysis

Call files recorded during the acoustic surveys were analysed using AnaScheme software, vers 1.0 [32], [33]. AnaScheme reads sound files recorded by Anabat detectors, and identifies bat pulses using a regional identification key; ours was built by BL, based on keys developed for Law and Chidel [14] and Hanspach et al. [34] (see Appendix S2, contact BL for further information). It included 14 species (Table 1), and of these only *Rhinolophus megaphyllus* was not recorded during the surveys. Calls of *N. geoffroyi* and *N. gouldi* cannot be reliably distinguished, therefore the two species were pooled as “*Nyctophilus* sp.”. This was also the case for *Vespadelus darlingtoni* (40–45 kHz) and *V. regulus* (40–45 kHz), which were pooled as “*Vespadelus darlingtoni*/*regulus*”. *V. regulus* is known to also produce a higher-frequency (HF) call (54–55 kHz) around large water courses in the field area (Law et al. 2002) so AnaScheme identified these separately as “*Vespadelus regulus* HF”. We set AnaScheme so that if >50% of pulses could not be definitively allocated to a single species because of low call quality, or because multiple species were calling at once, the file was identified as an “Unknown sp.”. All files identified as containing bat calls also were separately filtered for feeding buzzes, using a filter developed by BL. Any files flagged as containing feeding buzzes were then manually and audibly checked.

**Table 1:** Bat species recorded in the surveys.

	Remnants (118)			Fields (107)			Total (225)		
Species	C	A	F	C	A	F	C	A	F
Unknown sp.	116	57,490	6	105	17,202	30	221	74,692	36
*Vespadelus vulturnus*	108	2,881	33	96	3267	72	204	6,148	105
*Chalinolobus gouldii*	86	1,507	31	73	2344	164	159	3,851	195
*Mormopterus* sp. 4	72	730	11	71	1477	42	143	2,207	53
*Scotorepens greyii*	51	1,879	7	42	320	7	93	2,199	14
*Scotorepens balstoni*	56	511	13	49	318	28	105	829	41
*Mormopterus* sp. 2	33	279	20	41	354	5	74	633	25
*Tadarida australis*	40	246	0	50	233	1	90	479	1
*Vespadelus darlingtoni/regulus*	43	144	2	41	235	9	84	379	11
*Chalinolobus morio*	44	109	1	31	132	0	75	241	1
*Nyctophilus* sp.	28	52	2	43	138	3	71	190	5
*Vespadelus regulus* (HF)	22	59	3	26	55	1	48	114	4
*Chalinolobus picatus*	3	3	0	3	3	0	6	6	0
*Saccolaimus flaviventris*	1	1	0	0	0	0	1	1	0
*Rhinolophus megaphyllus*	0	0	0	0	0	0	0	0	0
Total		65,891	129		26,078	362		91,969	491

Species are listed in descending order according to total activity across all sites. “C” is “Count”, the number of survey points that each species was recorded at, with the total listed in parenthesis in the header row. “A” represents “Activity”, the number of calls recorded, and “F” represents “Feeding buzzes”. An expanded version of this list is supplied in Table S4.

Based on the above call analysis, we considered three bat responses at each survey point for each night: (1) species richness, the number of species identified each night, not including the ‘Unknown’ calls; (2) total activity, the number of files containing bat calls, irrespective of identification; and (3) feeding buzzes, an index of the number of files containing feeding activity, irrespective of identification.

### Data Analysis

**Do linear remnants and fields differ in habitat value for bats?** All analyses were conducted using ‘R’, vers 2.13.1 (http://www.r-project.org/). We first compared the three bat responses (species richness, activity, and feeding buzzes) between remnants and fields, by log-transforming the species richness and activity data, and running equal-variance t-tests. The feeding buzz data could not be transformed to fit a normal distribution, so we used a non-parametric Wilcox rank-sum test instead. To test for differences in bat species composition between land-use classes (canola crop, cereal crop, exotic pasture, native pasture and remnants), we used non-metric multidimensional scaling on the activity matrix of species (excluding *Chalinolobus picatus* and *Saccolaimus flaviventris*, rarely recorded) using the ‘metaMDS’ function in the ‘vegan’ package.

**Figure 3:**
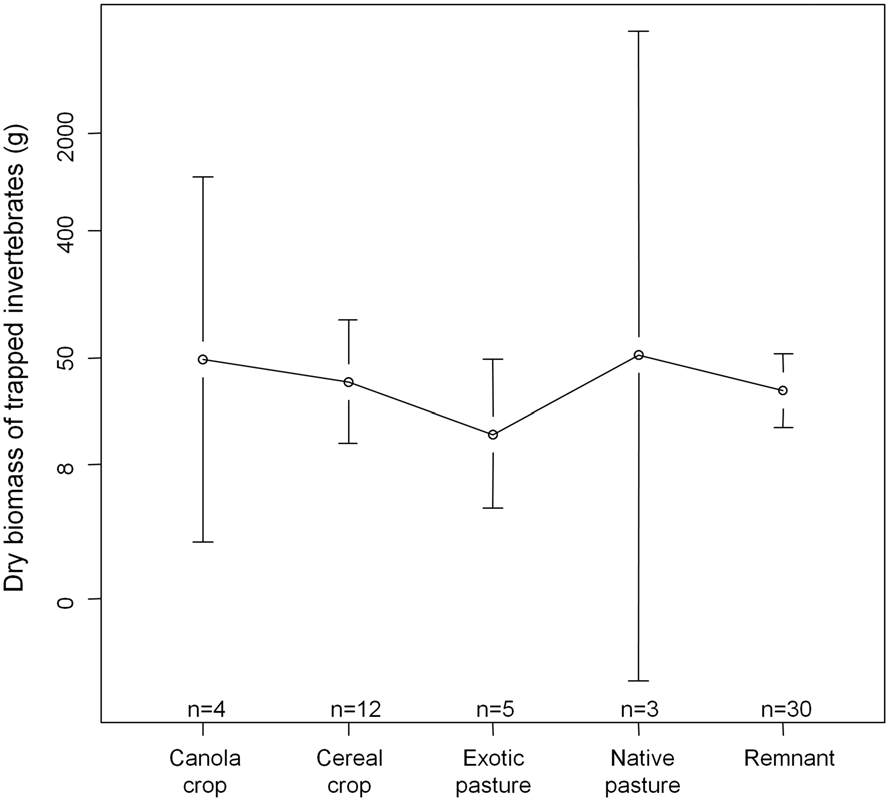
PCA biplot with loadings of the five vegetation measures on habitat components 1 and 2. Habitat component 1 separates sites according to condition, ranging from ‘intact’ (more negative scores) to ‘degraded’ (more positive scores) Habitat component 2 related to site structure: ‘shrub/grassland’ in the negative values to ‘grazed/cropped woodland’ in the positive values. Survey points are plotted according to the land use class that they occur within.

Fewer arthropod samples were collected than planned. High rainfall meant that some samples were washed away (14 of 98 samples), and trap number two may have been faulty, as it collected significantly smaller arthropod samples (18 samples, see Fig. S3). In addition, some traps appeared to not have switched on reliably, collecting few insects in some nights (<1.0 g dry biomass). In total, 54 samples were considered reliable and could be analysed (Fig. 3). Dry biomass from these samples was log-transformed, and we ran a one-way ANOVA to test for differences in arthropod biomass between land-use classes. For each of our bat responses from both remnants and fields, we used Spearman rank correlation to test whether the relationships with arthropod biomass were significant.

**What kinds of linear remnants are most important for bat conservation?** We used generalised linear mixed-effects models (GLMMs) to model the responses of bats (species richness, activity, feeding buzzes) in the linear remnants. Because bats are highly mobile, we were interested in the scale at which variables would impact on the responses. Therefore, we grouped our predictor variables according to whether they occurred locally to the trapping point, in the area directly adjacent (10 s to 100 s of metres), or within the wider landscape context (100 s to 1000 s of metres).

For ‘local’ effects, we used habitat components 1 and 2 from the PCA, as well as the width of the linear remnant, and the type or road running next to it (Table 2). The ‘adjacent’ variable used was the land use in the adjoining field. ‘Landscape’ variables included the distances of each survey point to the nearest natural water body or farm dam with a surface area >1 ha (‘distance to water’, see Appendix S1) and nearest conservation area (‘distance to conservation area’; based on data supplied by NSW Office of Environment and Heritage “Land Use: New South Wales”). Finally, ‘conditions’ variables accounted for weather, presence or absence of a black-light trap, and the survey period. Skewed explanatory variables were log-transformed prior to the analyses, and continuous variables were standardised to have a mean of zero and a standard deviation of one.

**Table 2:** Groups of explanatory variables used to construct the alternative generalised linear mixed models predicting bat species richness, activity and feeding (see Table 3, Table S3).

Variable group name	Variables in remnant models	Variables in field models
1. Local habitat “LOC”	Remnant width	Distance from the remnant
	Road type	Land use in the field
	Habitat component 1	Habitat component 1
	Habitat component 2	Habitat component 2
2. Adjacent habitat “ADJ”	Land use in adjacent field	Width of the adjacent remnant
		Adjacent road type
3. Landscape context “LSCP”	Distance to conservation area	Distance to conservation area
	Distance to water body	Distance to water body
4. Survey conditions “COND”	Presence/absence of rain	Presence/absence of rain
	Maximum wind speed	Maximum wind speed
	Maximum temperature	Maximum temperature
	Presence/absence of light trap	Presence/absence of light trap
	Survey period	Survey period

Combinations of these explanatory variable groups (local, adjacent, landscape, and conditions) resulted in 15 alternative models, and we also tested a 16^th^ ‘null model’ made up of random effects only, to determine if the explanatory variables predicted any more than our study design alone (Table S3). The random effect structure used in the models differed for each response, and this was based on visual inspection of the influence of each random effect (study site, survey point, and survey night) on responses, and also statistical methods outlined in Zuur et al. [35]. We used ‘study site’ as the random effect for the species richness data, because the survey point did not appear to influence the data. However, for feeding buzz data, the survey point did appear influential, and hence we used ‘study site/survey point’ in this case. There was evidence of overdispersion in the activity data, and to correct for this we added the random effect “night” (‘study site/survey point/night’). Each of the 16 alternative GLMMs were applied to each of the three bat response variables (species richness, activity, and feeding) assuming a Poisson distribution and using a log-link function in the ‘glmer’ function in the ‘lme4’ package for R.

For model selection we used an information-theoretic approach as implemented in the ‘AICcmodavg’ package. For each response we constructed 95% confidence tables, which list all models of the potential 16 tested with summed corrected Akaike weights (‘c*w_i_*’, which corrects for small sample sizes) ≥0.95. To pick a ‘final model’ which best explained the patterns in our data, we compared corrected Akaike’s Information Criteria (‘AICc’), log-likelihood (‘Log(*L*)’) and c*w_i_* for each of the models in the table. After choosing our final model, we judged which of the explanatory variables were having a strong influence by the magnitude of the coefficient estimate, visual inspection of plots, and also whether the 95% confidence intervals included zero.

**What kinds of ‘wildlife friendly’ measures make fields better habitat for bats?** Analysis for this question closely followed that described above – we again used GLMMs to predict the three bat responses, this time using the data collected in fields. Our 16 candidate models were the same as for the remnants, however some of the variables switched between groups to reflect the change of survey location. The ‘local’ variable group consisted of land use, the distance of the survey point from the remnant, and habitat components 1 and 2, and the ‘adjacent’ group contained the width of the adjacent remnant, and road type. The ‘landscape’ and ‘conditions’ groups remained the same (Table 2). The random effect structure for a given response and model selection was the same as described for the remnant analysis.

## Results

Across 228 detector nights and 2,475 survey hours, we recorded 1,193,152 sound files. Of these, 91,969 (7.7%) were confirmed as bat calls (403 passes/night), and 17,277 (19% of bat calls) could be identified. The majority of the calls (81%) were classified as ‘Unidentified’ because the key was conservative, and was designed to not mis-identify any calls for the sake of the species richness measures (as outlined in Appendix S2). Although the filter matched 3,031 files as containing feeding buzzes, only 491 files were confirmed as buzzes when manually checked (2.8% of bat calls). A total of 13 taxa were recorded (Table 1), and of these, *V. vulturnus* and *C. gouldii* were the most common, present at 98% and 91% of the survey points respectively. The two species listed as threatened were the least common, namely *C. picatus* (n = 6), and *S. flaviventris* (n = 1, Table S4). The species inventory appears to be reasonably complete, as confirmed by both trapping surveys in the study area and rarefaction analysis (Fig. S4).

### Do Linear Remnants and Fields Differ in Habitat Value for Bats?

**Figure 4:**
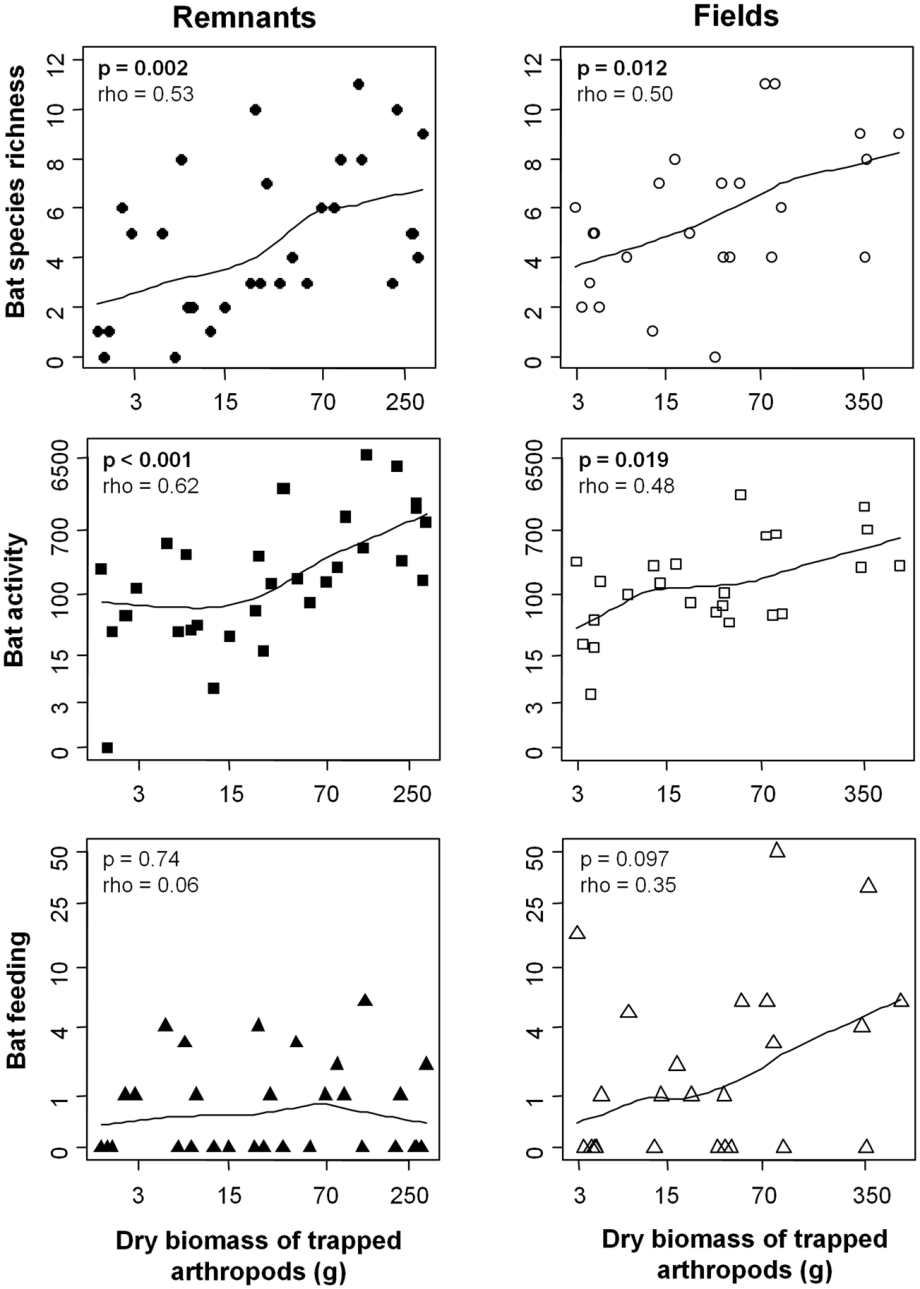
The relationship between bat responses and arthropod biomass, in both remnants and fields. Parameters from the Spearman-rank correlation analysis of each relationship are listed on the plots, and a smooth curve has been fitted for visualisation.

There were no significant differences between remnants and fields with regards to bat species richness (p = 0.434, t = 0.7842, df = 223, remnant mean = 5.02, field mean = 5.38) or the number of feeding buzzes recorded (p = 0.178, W = 6893, remnant mean = 1.1, field mean = 3.4). ). However, total bat activity in the remnants was double that of the fields (p = 0.044, t = −2.0283, df = 223, remnant mean = 609.84, field mean = 257.74). No clear differences in community composition were apparent between land use classes (Fig. S5). Arthropod biomass also did not significantly differ between land use classes (p = 0.603, df = 4, F = 0.688, Fig. 3). There were significant correlations between bat species richness, and also bat activity and arthropod biomass in both fields and remnants, but no such relationship was evident for feeding buzzes (Fig. 4).

### What Kinds of Linear Remnants are most Important for Bat Conservation?

**Figure 5:**
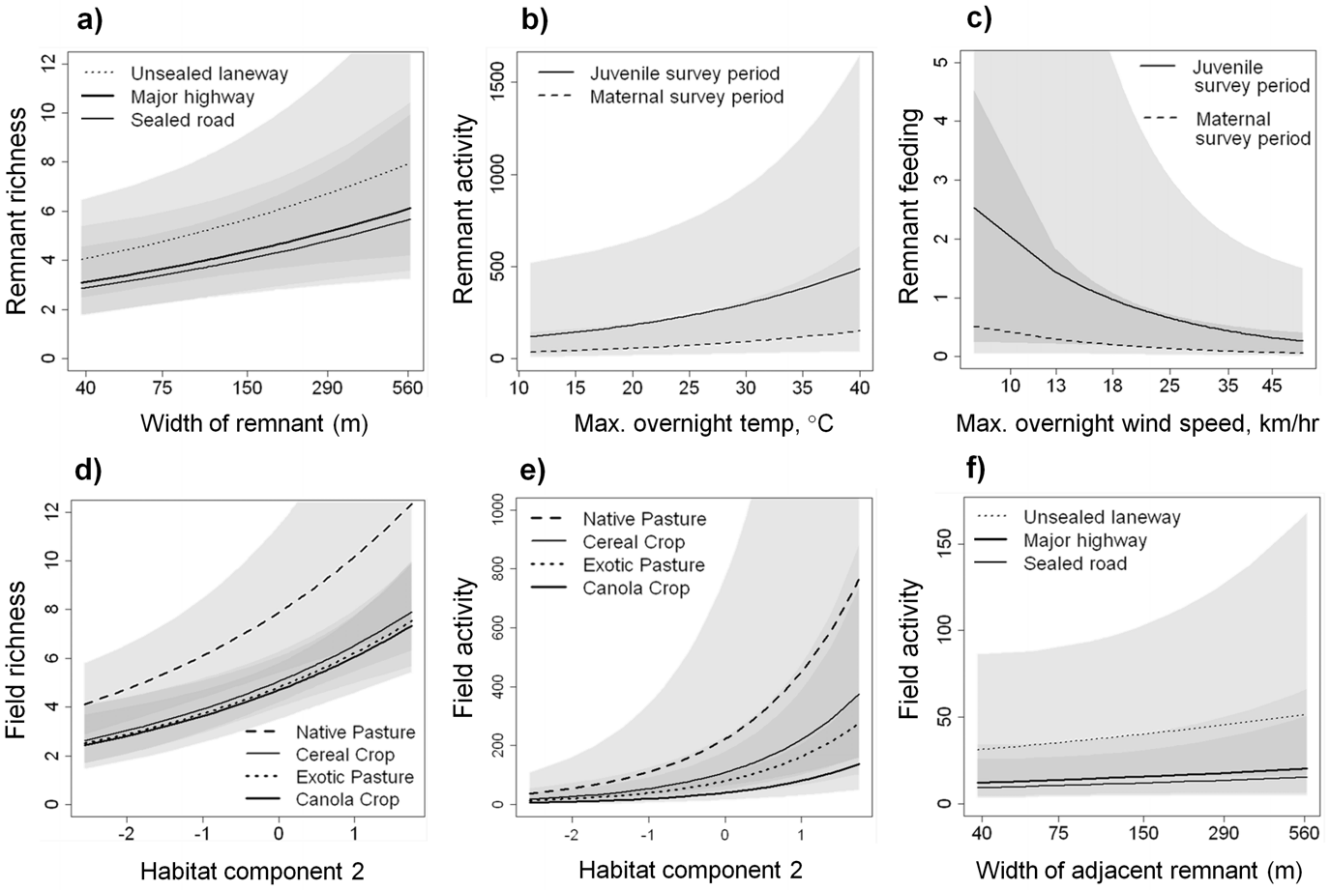
Influential predictor variables for bat responses. a) bat species richness in remnants, b) bat activity in remnants, c) bat feeding in remnants, d) bat species richness in fields, e) and f) bat activity in fields. More positive values of habitat component 2 indicate that a site has a structure which closer resembles a grazed or cropped woodland (mote trees, hollows and logs), as opposed to a shrub/grassland. Model parameters are listed in Tables 5 and 6). Semi-transparent polygons depict 95% confidence intervals.

The ‘adjacent’ variable group was ranked as the best predictor of bat species richness in the linear remnants (relative importance 0.95, Tables 3 and 4), but coefficient estimates for land use categories were of a low magnitude and the 95% confidence intervals included zero (Table 5), indicating they were not having a very strong effect. Because the ‘local’ variable group (relative importance 0.39, Table 4) also appeared to have a strong effect on species richness data, we selected the second-highest ranked model (‘local’ + ‘adjacent’; Table 3) for plotting and interpretation. Wider linear remnants were the most species rich, as were those that ran next to unsealed laneways and had a more intact vegetation structure (Table 5, Fig. 5a).

**Table 3:** 95% confidence tables resulting from analyses of bat responses in remnants and fields.

Response	Model no.	LOC	ADJ	LSCP	COND	AICc	c*w_i_*	Log(*L*)
Remnants – species richness	2		X			176.94	0.39	−82.06
	4^*^	X	X			177.13	0.35	−76.19
	9		X		X	179.46	0.11	−77.36
	5		X	X		180.89	0.05	−81.72
	11	X	X		X	181.48	0.04	−71.75
Remnants - activity	9^*^		X		X	747.01	0.84	−359.86
	12		X	X	X	750.60	0.14	−359.04
Remnants - feeding	9^*^		X		X	220.34	0.39	−97.79
	15				X	221.79	0.19	−102.22
	11	X	X		X	223.31	0.09	−92.67
	8	X			X	224.45	0.05	−97.42
	4	X	X			224.55	0.05	−99.90
	12		X	X	X	224.61	0.05	−97.37
	1	X				224.81	0.04	−103.73
	2		X			225.12	0.04	−106.15
	10			X	X	225.55	0.03	−101.72
	16 (NULL)					225.65	0.03	−109.72
	14	X	X	X	X	227.19	0.01	−91.75
Fields - species richness	1^*^	X				143.42	0.50	−62.93
	8	X			X	144.05	0.36	−56.94
	6	X		X		147.55	0.06	−62.55
	13	X		X	X	149.26	0.03	−56.80
Fields – activity	11^*^	X	X		X	599.60	0.72	−279.11
	8	X			X	602.06	0.21	−284.59
	14	X	X	X	X	604.82	0.05	−278.72
Fields - feeding	15^*^				X	276.05	0.83	−129.24
	10			X	X	279.95	0.12	−128.75

Table lists the variable groups included in the models, corrected Akaike’s Information Criteria (AICc), corrected Akaike Weights (c*w_i_*), and log-likelihood (Log(*L*)). Variable groups are described in Table 2, and model numbers are as defined in Table S3. Models denoted with asterices were used for plotting, and are further described in Tables 5 and 6.

**Table 4:** Relative importance of each variable group in each of the analyses.

Response variable	LOC	ADJ	LSCP	COND
Remnants - species richness	0.39	0.95	0.05	0.15
Remnants - activity	0.00	0.95	0.14	0.95
Remnants - feeding	0.24	0.63	0.09	0.81
Fields - species richness	0.95	0.00	0.09	0.39
Fields - activity	0.99	0.85	0.05	0.99
Fields - feeding	0.00	0.00	0.12	0.95

Relative importance was calculated by summing the corrected Akaike Weights (c*w_i_*) of every model in the 95% confidence tables that included the variable group of interest (Table 3).

**Table 5:** Model parameters predicting bat species richness, activity and feeding in remnants, showing the coefficient, standard error (SE), and lower and upper 95% confidence intervals (CI upp and CI low respectively) for each variable in the final model.

		RICHNESS				ACTIVITY				FEEDING			
Var. group	Term	Estimate	SE	CI low	CI upp	Estimate	SE	CI low	CI upp	Estimate	SE	CI low	CI upp
	Intercept	1.732	0.238	1.256	2.208	4.374	0.600	3.174	5.574	−1.670	0.796	−3.262	−0.078
Local habitat “LOC”	Remnant width	0.169	0.075	0.019	0.319								
	Road - Major	−0.263	0.214	−0.691	0.165								
	Road - Sealed	−0.338	0.156	−0.65	−0.026								
	Hab. comp. 1	−0.090	0.063	−0.216	0.036								
	Hab. comp. 2	−0.008	0.068	−0.144	0.128								
Adjacent habitat“ADJ”	Land use - Cereal crop	−0.058	0.203	−0.464	0.348	0.305	0.644	−0.983	1.593	−0.045	0.844	−1.733	1.643
	Land use - Exotic pasture	−0.088	0.197	−0.482	0.306	0.490	0.645	−0.8	1.78	−0.059	0.845	−1.749	1.631
	Land use - Native Pasture	−0.061	0.247	−0.555	0.433	0.309	0.757	−1.205	1.823	0.583	0.974	−1.365	2.531
Survey conditions “COND”	Wind speed					−0.216	0.143	−0.502	0.07	−0.381	0.139	−0.659	−0.103
	Temperature					0.320	0.156	0.008	0.632	−0.223	0.184	−0.591	0.145
	Rain - Present					−0.151	0.291	−0.733	0.431	0.412	0.312	−0.212	1.036
	Light trap –Present					−0.292	0.207	−0.706	0.122	−0.228	0.208	−0.644	0.188
	Survey period - juvenile					0.859	0.311	0.237	1.481	1.370	0.451	0.468	2.272

For both bat activity and feeding in the remnants, the ‘adjacent’ and ‘conditions’ variable groups constituted the highest-ranked models (Table 3), however, once again land use did not appear to have a very strong effect (Table 5). Both activity and feeding levels were higher in the juvenile survey period (Fig. 5b and 5c), though temperature was a better predictor of activity data, and wind speed of feeding data. The large number of models included in the 95% confidence table for feeding in remnants (Table 3), which includes Model 16 (the ‘null model’), indicated that there was a very high degree of uncertainty in predicting bat feeding behaviour.

### What Kinds of ‘Wildlife Friendly’ Measures Make Fields Better Habitat for Bats?

The highest-ranked model for bat species richness in fields included the ‘local’ variable group only, with a relative importance of 0.95 (Tables 3 and 4). Fields containing native, unimproved pastures supported the most species-rich communities of bats compared with other land use categories (exotic pasture, canola, or cereal crop, Fig. 5d), and a positive effect of habitat component 2 indicated that bat species richness increased with a greater number of trees, number of hollows and log volume (Fig. 2, 5d). A large number of variables strongly predicted for bat activity in the fields – the ‘local’, ‘adjacent’, and ‘conditions’ groups were all included in the highest-ranked model (Table 3). Again, there was a positive effect of native pastures, as well as those with higher values of habitat component 2 (‘grazed/cropped woodlands’, Table 6, Fig. 5e). In concordance with our findings from the remnants, we recorded marginally higher bat activity in fields next to unsealed laneways (Fig. 5f). Finally, only “conditions” affected the number of feeding buzzes recorded in fields, as the model of this variable group alone was very highly weighted (c*w_i_* = 0.83, Table 3). The presence of a light trap in particular led to higher levels of feeding activity (Table 6).

**Table 6:** Model parameters predicting bat species richness, activity and feeding in fields, showing the coefficient, standard error (SE), and lower and upper 95% confidence intervals (CI upp and CI low respectively) for each variable in the final model.

		RICHNESS				ACTIVITY				FEEDING			
Var. group	Term	Estimate	SE	CI low	CI upp	Estimate	SE	CI low	CI upp	Estimate	SE	CI low	CI upp
	Intercept	1.546	0.146	1.254	1.838	3.693	0.470	2.753	4.633	−0.821	0.432	−1.685	0.043
Local habitat “LOC”	Land use - Cereal crop	0.075	0.160	−0.245	0.395	1.002	0.401	0.2	1.804				
	Land use - Exotic pasture	0.026	0.170	−0.314	0.366	0.694	0.422	−0.15	1.538				
	Land use - Native Pasture	0.518	0.260	−0.002	1.038	1.712	0.687	0.338	3.086				
	Hab. comp. 1	−0.105	0.064	−0.233	0.023	−0.214	0.156	−0.526	0.098				
	Hab. comp. 2	0.256	0.064	0.128	0.384	0.706	0.149	0.408	1.004				
	Distance into field	0.053	0.047	−0.041	0.147	−0.010	0.108	−0.226	0.206				
Adjacent habitat“ADJ”	Road - Major					−0.932	0.450	−1.832	−0.032				
	Road - Sealed					−1.198	0.347	−1.892	−0.504				
	Remnant width					0.124	0.142	−0.16	0.408				
Survey conditions “COND”	Wind speed					−0.063	0.107	−0.277	0.151	−0.230	0.122	−0.474	0.014
	Temperature					−0.085	0.127	−0.339	0.169	0.205	0.179	−0.153	0.563
	Rain - Present					−0.287	0.236	−0.759	0.185	0.089	0.158	−0.227	0.405
	Light trap –Present					0.180	0.196	−0.212	0.572	0.611	0.148	0.315	0.907
	Survey period - juvenile					1.575	0.230	1.115	2.035	−0.476	0.377	−1.23	0.278

The ‘landscape’ variable group was not included in any of the final models, so is not listed here.

## Discussion

### Do Linear Remnants and Fields Differ in Habitat Value for Bats?

Surprisingly, the only detectable difference between linear remnants and fields was higher bat activity in remnants, and was not due to a greater availability of prey (Fig. 3). An alternative explanation could be that there were more active roosts in the remnants; if we assume that bats prefer to roost in trees >70 cm in diameter with hollows (based on [36], [37]), remnants in our study supported on average 6.53 (±0.67 SE) potential roost trees per hectare, compared with 2.22 (±0.35 SE) in fields. This figure for fields within the study region is also likely to be an over-estimation, because we only conducted surveys in fields with scattered trees, and these individual trees are uncommonly used as roosts [37], [38].

Perhaps even more surprisingly, we did not detect differences in community composition between land uses (Fig. S5). This may be because the woodland communities that naturally occur in this region are quite open and thus do not preclude foraging by open-area species, and clutter-tolerant species are not necessarily limited to foraging in cluttered areas [39]. Analyses relating to the requirements of individual species will be necessary to determine more subtle effects of land use on the occupancy of remnants and fields by different bat fauna.

### What Kinds of Linear Remnants are most Important for Bat Conservation?

Our surveys indicated that wider remnants composed of intact, structurally complex vegetation form the best habitat for diverse bat communities (Fig. 5a, Table 5), which is very much in line with conventional conservation wisdom [40]. Remnants next to sealed roads supported lower bat species richness than those next to unsealed laneways. However, there was not a great difference between single-lane roads and multi-lane highways (Fig. 5a), which might suggest that it is not the level of traffic itself that is deterring bats, but rather the nature of the bitumen surface. Potential causes of lower richness with the sealed road surface need to be explored further. In their study of the BAB3 motorway in Germany, Kerth and Melber [41] found that a ‘clutter-tolerant’ bat species was more vulnerable to the effects of the road than an open-area adapted bat species, so it would be valuable to determine the role that ecomorphology plays in these circumstances.

### What Kinds of ‘Wildlife Friendly’ Measures Make Fields Better Habitat for Bats?

Scattered trees in fields, and the structures associated with them such as logs and hollows, were found to be important in maintaining high bat activity, most likely because they provide a source of forage and shelter from predators [42], [43]. The importance of this finding needs to be reinforced to land managers, because scattered trees are being lost from farming landscapes globally [44]. This is especially the case in cropping environments, where trees compete for water and nutrients and are an obstacle for large equipment [45]. However, these fields are also the most likely to benefit from bat predation services on pests.

In spite of uncertainty regarding the value of more ‘wildlife friendly’ land uses to bats [12], we found a clear positive effect of unimproved native pastures on both bat species richness and activity in fields. This cannot be explained by prey availability alone (Fig. 3), and although lower pesticide inputs in native pastures should benefit arthropod diversity, higher nutrient loads in more intensively managed areas can also lead to outbreaks in a small number of herbivorous insect species [46]. Given this, the habitat native pastures provide by forming a ‘softer’ matrix is also likely to be playing a role.

### How can Agricultural Landscapes Best be Managed for Bat Conservation?

Plausible management goals in agricultural landscapes are to maximise bat richness in remnants (for the sake of conservation) and bat activity in fields (for pest control purposes). For these goals, based on our findings, the retention of natural structures such as trees and an understorey not strongly modified by grazing impacts or cropping is important. Our results imply that conservation actions are likely to be more successful if conducted in areas close to unsealed, rather than sealed roads. Unfortunately, it is less clear how to manage for bat feeding specifically, because responses were not as strong and mostly related to conditions during surveys. However, other studies in agricultural and urban areas have found higher rates of feeding over more fertile geologies [25], [47], suggesting that conserving remnant vegetation in productive parts of the landscape is important for bats. The juvenile survey period also saw a considerable increase in activity, so structures that allow for successful breeding close to fields, such as linear remnants and large retained trees within them, need to be maintained.

Contrary to our expectations, the bats in our study did not respond to the proximity of water or protected areas. Given the mobility of bats, all of our survey points may have been within easy commuting distance from water or roosts, or these may have been accessed in the linear remnants themselves. We may also not have found an effect of distance to water because of the high rainfall during the survey period, which resulted in free-standing water being present across much of the landscape (Appendix S1, Fig. S6). However, in drier years streams in remnants and dams in fields are likely to form important resources for bats [11], [48].

It should be noted that a considerable proportion of the landholders we spoke with indicated they were not aware that bats used their fields for either roosting or foraging. This further strengthens the case for better communicating the persistence of these cryptic taxa in agricultural landscapes, especially given the positive indirect impact bats are likely to have on crop yield through pest predation [8], [48]. Furthermore, the two types of areas surveyed in this study (linear remnants and fields) have historically been managed as separate entities by separate actors, yet variables relating to areas adjacent to the survey points were strong predictors of bat responses in many of our models (Table 3). A more integrated approach to landscape planning and management, which takes into account not only the individual features or fields but also the surrounding landscape [49], is therefore required.

This study has revealed some key features that can be manipulated to conserve bats in agricultural landscapes, which should be further incorporated into AES. In particular, linear elements can support high bat activity if managed appropriately. Moreover, by harbouring bat communities and having high edge to area ratios, linear elements have the potential to provide pest predation ecosystem services to a greater number of fields than more remote or isolated reserves alone. Finally, there appears to be no clear-cut answer as to whether “wildlife friendly farming” (the integration of wildlife-friendly features into fields) or “land sparing” (the protection of designated areas) is preferable for bats. So far most arguments for “land sparing” have focused on birds [5], [6]. However in our study system, we found evidence that the integration of both approaches could be useful because both conditions in fields and linear remnants influenced bat communities.

## Supporting Information

Figure S1 **The eight stages of** ***Eucalyptus*** **senescence, taken from Rayner (2008).** (1) Immature tree, branches upright, (2) mature, adult tree, branches spread and intact with healthy crown, (3) mature tree with signs of senescence, some large broken branches, crown thinning (<50%), (4) live adult tree, largely bare, but small patches of canopy or areas of regrowth, (5) dead stag with majority of branches (>50%) intact, (6) dead stag with <50% branches remaining, (7) upright, dead stag with no major branches remaining, and (8) broken or cut stump.
(TIF)

Figure S2 **Differences in the mean levels of the two habitat components between the different land use classes.** a) habitat component 1, and b) habitat component 2. Land use class contrasts are shown on the y-axes.
(TIF)

Figure S3 **Mean dry biomass of nocturnal arthropod samples collected in each of the black-light traps.** Error bars represent 95% confidence intervals, and sample sizes are listed below each of the plotting points.
(TIF)

Figure S4 **Rarefaction curve, showing the accumulation of species with each additional acoustic survey.**
(TIF)

Figure S5 **Arrangement of survey points of different land-use classes on three axes, from non-metric multidimensional scaling (NMDS).** Stress = 0.17. A subsequent Analysis of Similarities test (ANOSIM), run using the ‘anosim’ function in the ‘vegan’ package in R, indicated that there was significant greater between- than within- group variation, though these differences were minor (R = 0.06657, p = 0.0136).
(TIF)

Figure S6 **Free-standing water and pools present in many of the remnant and field sites.**
(TIF)

Table S1 Descriptions of the seven types of bark encountered in field surveys of *Eucalyptus* trees, with example species.
(DOC)

Table S2 Loadings of variables on habitat components 1 and 2, from the principle components analysis of vegetation measures taken from a 1 ha area around each survey point.
(DOC)

Table S3 Combination of variable groups used in the 16 alternative generalised linear mixed models, used to predict bat species richness, activity and feeding.
(DOC)

Table S4 Expanded list of species recorded in each of the land use classes.
(DOC)

Appendix S1 **The importance of water and riparian areas.**
(DOC)

Appendix S2 **Bat call analysis in AnaScheme.**
(DOC)

Appendix S3 **Data exploration and potential correlation of fixed effects.**
(DOC)
