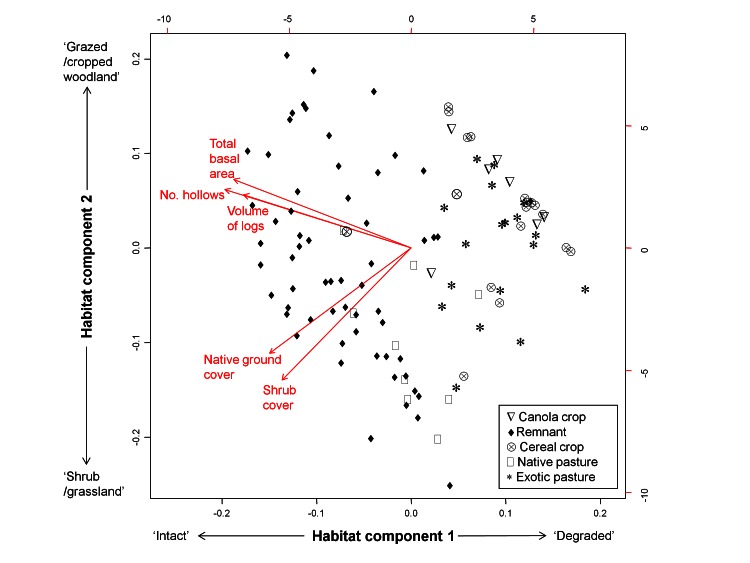# Correction: Bats in a Farming Landscape Benefit from Linear Remnants and Unimproved Pastures

**DOI:** 10.1371/annotation/373d69c1-9931-4c5a-a24d-4fe664c08ddc

**Published:** 2013-05-23

**Authors:** Pia E. Lentini, Philip Gibbons, Joern Fischer, Brad Law, Jan Hanspach, Tara G. Martin

Figures 2 and 3 had their placement reversed in the article.

The correct figures are:

Figure 2: 

**Figure:**
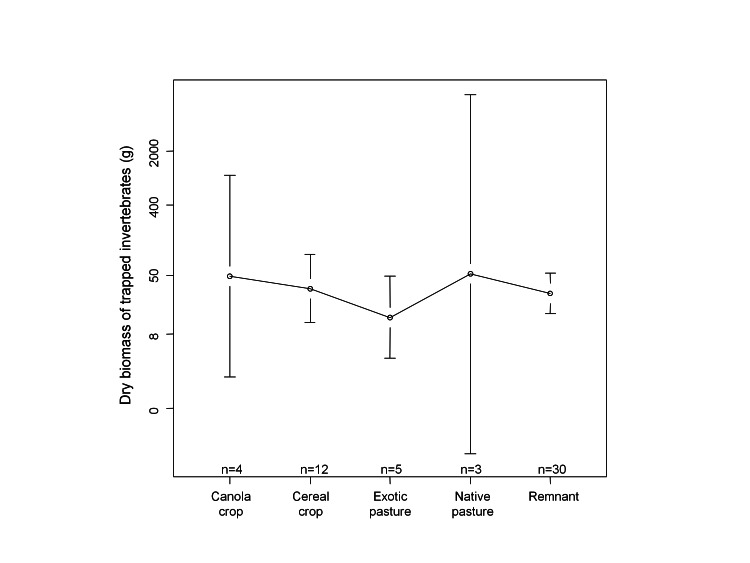


Figure 3: 

**Figure:**