# Supplementary figures and images for: Bats in a Farming Landscape Benefit from Linear Remnants and Unimproved Pastures
Source: PLoS One. 2012 Nov 14;7(11):e48201. doi: 10.1371/journal.pone.0048201 (PMC3498260; doi:10.1371/journal.pone.0048201)

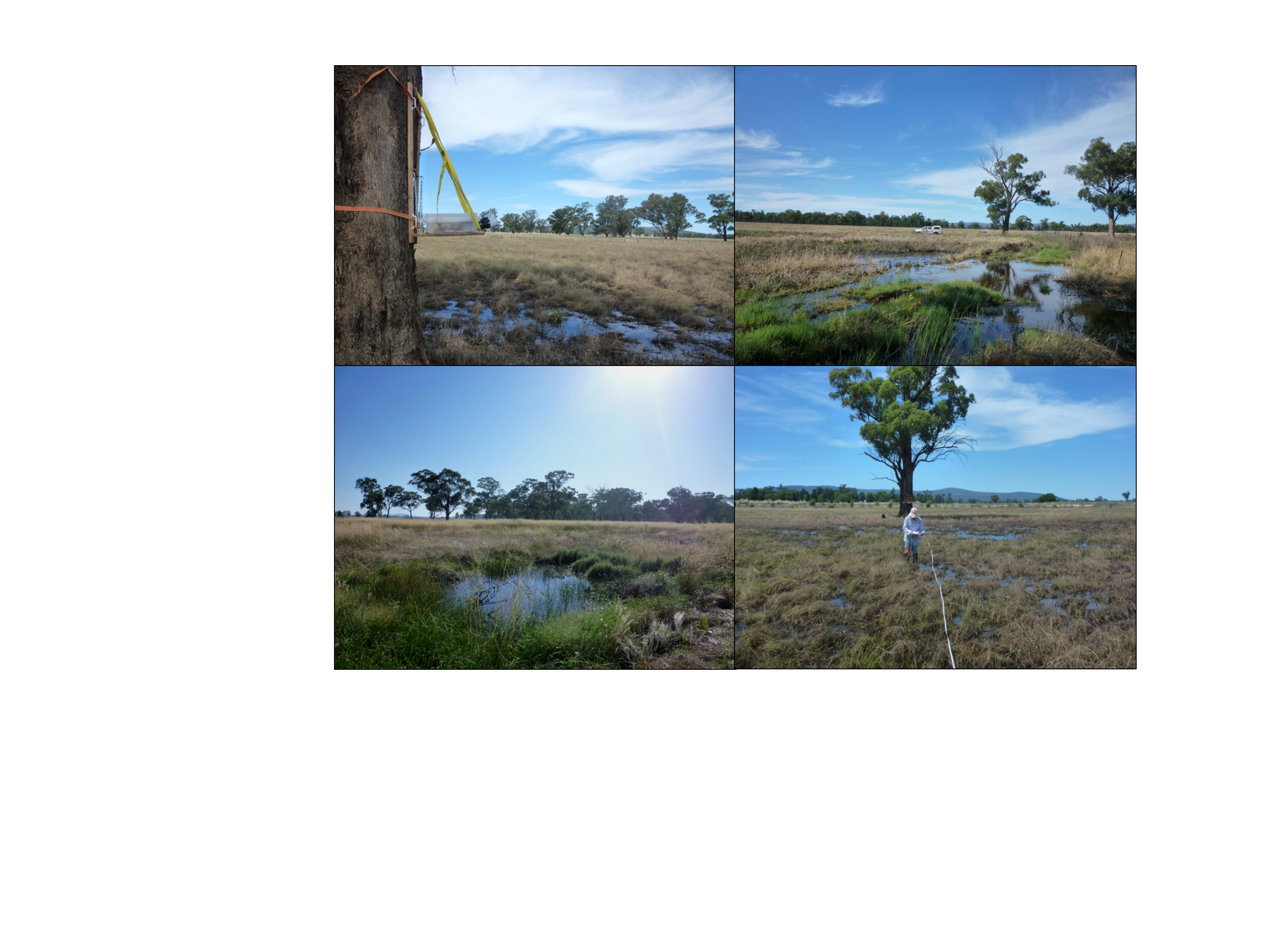

Supplement: Figure S6 — Free-standing water and pools present in many of the remnant and field sites. (TIF) [file pone.0048201.s006.tif]
