# Supplementary material for: Bats in a Farming Landscape Benefit from Linear Remnants and Unimproved Pastures
Source: PLoS One. 2012 Nov 14;7(11):e48201. doi: 10.1371/journal.pone.0048201 (PMC3498260; doi:10.1371/journal.pone.0048201)
Supplement: Table S1 — Descriptions of the seven types of bark encountered in field surveys of Eucalyptus trees, with example species. (DOC) [file pone.0048201.s007.doc]

Table S1. Descriptions of the seven types of bark encountered in field surveys of *Eucalyptus* trees, with example species.

| **Bark type** | **Description** | **Typical groups** | **Example species** |
| --- | --- | --- | --- |
| **Smooth** | Smooth bark over entire tree | Mallees | *Eucalyptus rubida, E. rosii* |
| **Flaked** | Annual bark shedding, residual bark in flakes, underlying bark is smooth | Red gums | *E. mannifera,*  *E. blakelyi* |
| **Rough base** | Smooth bark over branches and upper trunk, rough or ribbon bark collects at trunk base | Ribbon gums | *E. melliodora* |
| **Fibrous** | Long-fibred, partially furrowed, spongy bark that can be stripped manually in long pieces | Stringy barks | *E. macrorhyncha* |
| **Sub-fibrous** | Bark fibres of short-medium length, narrow longitudinal fissures, possibly with shaggy base | Box species | *E. microcarpa,*  *E. populnea,*  *E. albens* |
| **Furrowed** | Bark is thick, hard, widely furrowed and retained on tree | Ironbarks | *E. sideroxylon* |
| **Tessalated** | Dead bark retained on tree in small, short-fibred plates/tiles | Bloodwoods | *E. gummifera* |
