# Supplementary material for: Bats in a Farming Landscape Benefit from Linear Remnants and Unimproved Pastures
Source: PLoS One. 2012 Nov 14;7(11):e48201. doi: 10.1371/journal.pone.0048201 (PMC3498260; doi:10.1371/journal.pone.0048201)
Supplement: Table S2 — Loadings of variables on habitat components 1 and 2, from the principle components analysis of vegetation measures taken from a 1 ha area around each survey point. (DOC) [file pone.0048201.s008.doc]

Table S2. Loadings of variables on habitat components 1 and 2, from the principle components analysis of vegetation measures taken from a 1 ha area around each survey point.

| **Covariate** | **Loadings of habitat component 1** | **Loadings of habitat component 2** |
| --- | --- | --- |
| Number of trees with hollows | -0.514 | 0.294 |
| Total basal area of trees | -0.490 | 0.347 |
| Volume of logs (m^3^) | -0.463 | 0.268 |
| Per cent ground cover which is native | -0.392 | -0.531 |
| Per cent cover of shrubs | -0.357 | -0.663 |
