# Supplementary material for: Bats in a Farming Landscape Benefit from Linear Remnants and Unimproved Pastures
Source: PLoS One. 2012 Nov 14;7(11):e48201. doi: 10.1371/journal.pone.0048201 (PMC3498260; doi:10.1371/journal.pone.0048201)
Supplement: Table S3 — Combination of variable groups used in the 16 alternative generalised linear mixed models, used to predict bat species richness, activity and feeding. (DOC) [file pone.0048201.s009.doc]

Table S3. Combination of variable groups used in the 16 alternative generalised linear mixed models, used to predict bat species richness, activity and feeding. An ‘X’ indicates that a variable group was included in the model.

| **Model no.** | **Local habitat “LOC”** | **Adjacent habitat**  **“ADJ”** | **Landscape context**  **“LSCP”** | **Survey conditions**  **“COND”** | **Random effects(s)** |
| --- | --- | --- | --- | --- | --- |
| **1** | X |  |  |  | X |
| **2** |  | X |  |  | X |
| **3** |  |  | X |  | X |
| **4** | X | X |  |  | X |
| **5** |  | X | X |  | X |
| **6** | X |  | X |  | X |
| **7** | X | X | X |  | X |
| **8** | X |  |  | X | X |
| **9** |  | X |  | X | X |
| **10** |  |  | X | X | X |
| **11** | X | X |  | X | X |
| **12** |  | X | X | X | X |
| **13** | X |  | X | X | X |
| **14** | X | X | X | X | X |
| **15** |  |  |  | X | X |
| **16 (null)** |  |  |  |  | X |
