# Supplementary material for: Bats in a Farming Landscape Benefit from Linear Remnants and Unimproved Pastures
Source: PLoS One. 2012 Nov 14;7(11):e48201. doi: 10.1371/journal.pone.0048201 (PMC3498260; doi:10.1371/journal.pone.0048201)
Supplement: Table S4 — Expanded list of species recorded in each of the land use classes. (DOC) [file pone.0048201.s010.doc]

Table S4. Expanded list of species recorded in each of the land use classes. “C” is “Count”, the number of survey points that each species was recorded at, with the listed in parenthesis in the header row. “A” represents “Activity”, the total number of calls recorded, and “F” represents “Feeding buzzes”.

|  | **Remnant** | | | **Cereal crop** | | | **Exotic pasture** | | | **Canola crop** | | | **Native pasture** | | | **Total** | | |
| --- | --- | --- | --- | --- | --- | --- | --- | --- | --- | --- | --- | --- | --- | --- | --- | --- | --- | --- |
|  | **(118)** | | | **(39)** | | | **(37)** | | | **(15)** | | | **(16)** | | | **(225)** | | |
| **Species** | C | A | F | C | A | F | C | A | F | C | A | F | C | A | F | C | A | F |
| Unknown sp. | 116 | 57,490 | 6 | 39 | 13,351 | 15 | 35 | 2,312 | 4 | 15 | 841 | 7 | 16 | 698 | 4 | 221 | 74,692 | 36 |
| *Vespadelus vulturnus* | 108 | 2,881 | 33 | 36 | 909 | 17 | 31 | 1,500 | 37 | 13 | 330 | 8 | 16 | 528 | 10 | 204 | 6,148 | 105 |
| *Chalinolobus gouldii* | 86 | 1,507 | 31 | 23 | 764 | 99 | 22 | 940 | 17 | 15 | 524 | 34 | 13 | 116 | 14 | 159 | 3,851 | 195 |
| *Mormopterus* sp. 4 | 72 | 730 | 11 | 25 | 653 | 25 | 21 | 467 | 11 | 12 | 188 | 3 | 13 | 169 | 3 | 143 | 2,207 | 53 |
| *Scotorepens greyii* | 51 | 1,879 | 7 | 20 | 112 | 2 | 10 | 102 | 2 | 3 | 36 | 3 | 9 | 70 | 0 | 93 | 2,199 | 14 |
| *Scotorepens balstoni* | 56 | 511 | 13 | 17 | 98 | 13 | 14 | 151 | 2 | 8 | 41 | 8 | 10 | 28 | 5 | 105 | 829 | 41 |
| *Mormopterus* sp. 2 | 33 | 279 | 20 | 21 | 182 | 2 | 7 | 19 | 1 | 5 | 14 | 0 | 8 | 139 | 2 | 74 | 633 | 25 |
| *Tadarida australis* | 40 | 246 | 0 | 21 | 94 | 0 | 15 | 32 | 1 | 6 | 26 | 0 | 8 | 81 | 0 | 90 | 479 | 1 |
| *Vespadelus darlingtoni/regulus* | 43 | 144 | 2 | 15 | 31 | 1 | 14 | 153 | 6 | 6 | 36 | 2 | 6 | 15 | 0 | 84 | 379 | 11 |
| *Chalinolobus morio* | 44 | 109 | 1 | 14 | 75 | 0 | 9 | 39 | 0 | 3 | 5 | 0 | 5 | 13 | 0 | 75 | 241 | 1 |
| *Nyctophilus* sp. | 28 | 52 | 2 | 12 | 47 | 2 | 16 | 57 | 0 | 5 | 12 | 1 | 10 | 22 | 0 | 71 | 190 | 5 |
| *Vespadelus regulus* (HF) | 22 | 59 | 3 | 12 | 34 | 0 | 4 | 9 | 1 | 2 | 2 | 0 | 8 | 10 | 0 | 48 | 114 | 4 |
| *Chalinolobus picatus* | 3 | 3 | 0 | 0 | 0 | 0 | 2 | 2 | 0 | 0 | 0 | 0 | 1 | 1 | 0 | 6 | 6 | 0 |
| *Saccolaimus flaviventris* | 1 | 1 | 0 | 0 | 0 | 0 | 0 | 0 | 0 | 0 | 0 | 0 | 0 | 0 | 0 | 1 | 1 | 0 |
| *Rhinolophus megaphyllus* | 0 | 0 | 0 | 0 | 0 | 0 | 0 | 0 | 0 | 0 | 0 | 0 | 0 | 0 | 0 | 0 | 0 | 0 |
| Total |  | 65,891 | 129 |  | 16,350 | 176 |  | 5,783 | 82 |  | 2,055 | 66 |  | 1,890 | 38 |  | 91,969 | 491 |
