# Supplementary material for: Bats in a Farming Landscape Benefit from Linear Remnants and Unimproved Pastures
Source: PLoS One. 2012 Nov 14;7(11):e48201. doi: 10.1371/journal.pone.0048201 (PMC3498260; doi:10.1371/journal.pone.0048201)
Supplement: Appendix S1 — The importance of water and riparian areas. (DOC) [file pone.0048201.s011.doc]

**Appendix S1 – The importance of water and riparian areas**

Many previous studies have found water resources, both natural and anthropogenic, to be important for bats [1-3]. Four of the 32 remnants in this study could be classified as ‘riparian’, in they had a small stream or creek (~2m wide) running through them. These four remnants did not support higher bat species richness compared with non-riparian remnants (5.2±0.54, cf 4.95±0.29), or bat activity (373.13±157.62, cf 625.68±145.8), or feeding buzzes (0.6±0.25, cf 1.18±0.25).

Using a GIS, we measured two separate distances for each survey point to water – the first was to any form of mapped water (streams, rivers, farm dams, or irrigation channels), irrespective of their size. The second was the distance to any natural water body or large farm dam, >1 ha in surface area. The two measures were highly correlated, and as we could only include one in our modelling, both were plotted against our bat responses. The second measure appeared to fit the data better, so we used this in our analyses. It is likely that neither the ‘riparian’ nature of the remnants, nor the distance to water, had a strong effect on the outcomes of our surveys because, at the time, water was abundant across much of the landscape. As noted in our Methods section, there was high rainfall in the summer that our surveys took place, so not only were rivers, streams and dams full or overflowing, but many fields were ankle-deep in free-standing water (Fig. S6). The extent of this free-standing water was quantified during the habitat surveys, as part of the ground-cover transects. However, if emergent vegetation was present at each point, this was recorded and ‘native’ or ‘non-native’ instead of ‘water’, so not enough ‘water’ data points were recorded to be meaningful in the analyses. Finally, we trialled adding the elevation of each of the survey points, to act as a proxy for the patterns of water accumulation across the landscape, to our models, but this did not strengthen them. We therefore concluded that, in years with very high rainfall, water resources are of lesser importance to bat communities than other factors, such as roost or forage availability.

**References**

1. Law BS, Anderson J, Chidel M (1998) A bat survey in State Forests on the south-west slopes region of New South Wales with suggestions of improvements for future surveys. Australian Zoologist 30: 467-479.

2. Wickramasinghe LP, Harris S, Jones G, Vaughan N (2003) Bat activity and species richness on organic and conventional farms: impact of agricultural intensification. Journal of Applied Ecology 40: 984-993.

3. Lundy M, Montgomery I (2010) Summer habitat associations of bats between riparian landscapes and within riparian areas. European Journal of Wildlife Research 56: 385-394.
