# Supplementary material for: Bats in a Farming Landscape Benefit from Linear Remnants and Unimproved Pastures
Source: PLoS One. 2012 Nov 14;7(11):e48201. doi: 10.1371/journal.pone.0048201 (PMC3498260; doi:10.1371/journal.pone.0048201)
Supplement: Appendix S2 — Bat call analysis in AnaScheme. (DOC) [file pone.0048201.s012.doc]

**Appendix S2 - Bat call analysis in AnaScheme**

Call files recorded during the acoustic surveys were analysed using AnaScheme software, vers 1.0 [1,2]. AnaScheme reads sound files recorded by Anabat detectors, and models individual bat search-phase pulses using regression analysis. Pulses are then identified, using a regional identification key; ours was built by BL, and was based on keys developed for Law & Chidel [3] and Hanspach *et al.* [4]. The key was tested on a library of 78 reference calls collected from the study region during pilot trapping surveys, and was found to correctly identify 72.4% of calls, 27.6% were deemed “Unknown”, and none were identified incorrectly. Our key included 14 species (Table 1), and of these only *Rhinolophus megaphyllus*, a cave-roosting bat which occurs to the eastern end of the study region, was not recorded during the surveys. Calls of *N. geoffroyi* and *N. gouldi* cannot be reliably distinguished, therefore the two species were pooled to form the “*Nyctophilus* sp.” complex. This was also the case for *Vespadelus darlingtoni* (40-45 kHz) and *V. regulus* (40-45 kHz), which were pooled as “*Vespadelus darlingtoni*/*regulus*”. *V. regulus* is known to also produce a higher-frequency (HF) call (54-55 kHz) around large water courses in the field area [3], so AnaScheme identified these separately as “*Vespadelus regulus* HF”.

We set AnaScheme to identify call files only when a minimum of three pulses, and >50 % of the pulses within a pass, were classified as the same species. We set AnaScheme so that if >50 % of pulses could not be definitively allocated to a single species because of low call quality, or because multiple species were calling at once, the file was identified as an “Unknown sp.”. Calls from the surveys which were identified as *Chalinolobus picatus* or *Saccolaimus flaviventris*, which are listed as threatened in the region, were manually checked, as were those of *Vespadelus regulus* “HF”. All files which AnaScheme identified to contain bat calls were then separately filtered for feeding buzzes, using a filter developed by BL that recognises short sequences of steep linear calls produced in rapid repetition, which typifies feeding buzzes. Any files flagged as containing feeding buzzes were also manually and audibly checked to exclude non-feeding buzzes using Anabat 6 software (vers 6.3, Chris Corben, [www.hoarybat.com](http://www.hoarybat.com/)).

**References**

1. Gibson M, Lumsden L (2003) The Anascheme automated bat call identification system. The Australasian Bat Society Newsletter 20: 24-26.

2. Adams MD, Law BS, Gibson MS (2010) Reliable automation of bat call identification for eastern New South Wales, Australia, using classification trees and AnaScheme software. Acta Chiropterologica 12: 231-245.

3. Law BS, Chidel M (2006) Eucalypt plantings on farms: Use by insectivorous bats in south-eastern Australia. Biological Conservation 133: 236-249.

4. Hanspach J, Fischer J, Ikin K, Stott J, Law BS (in press) Using trait-based filtering as a predictive framework for conservation: A case study of bats in southeastern Australia. Journal of Applied Ecology.
