# Supplementary material for: Bats in a Farming Landscape Benefit from Linear Remnants and Unimproved Pastures
Source: PLoS One. 2012 Nov 14;7(11):e48201. doi: 10.1371/journal.pone.0048201 (PMC3498260; doi:10.1371/journal.pone.0048201)
Supplement: Appendix S3 — Data exploration and potential correlation of fixed effects. (DOC) [file pone.0048201.s013.doc]

**Appendix S3 - Data exploration and potential correlation of fixed effects**

Many habitat measures were taken at each survey point, and we were limited in the number of explanatory variables we could include in our models. Some of the bat species which occur in our study region roost under decorticating bark [1], and trees in later stages of senescence are more likely to have developed hollows [2]. It is also known that some bat species are sensitive to high levels of habitat ‘clutter’ [3,4]. For these reasons, we suspected that variables relating to trees would be most important for bats, so we first trialled a Principle Component Analysis with the following variables: tree species, stage of senescence, the number of tree stems, and bark types. However, when the components resulting from this ordination were plotted against the bat responses, it appeared that they had little power in explaining the observed patterns. We therefore repeated the process with measures that we felt reflected the degree of anthropogenic disturbance at each site, and habitat structure, based on measurements commonly incorporated into condition assessments for these woodlands [5,6]. These were the variables presented in the final analysis: the total basal area of trees, the number of trees with hollows, the volume of logs, the percent of the ground cover which was native, and the percent cover of shrubs across the 1 ha site. The two new variables produced from this ordination, “habitat component 1” and “habitat component 2”, appeared to be more effective in explaining patterns in our bat responses.

We checked for correlation between all pairs of explanatory variables using the ‘cor’ function in R, and by visually inspecting pairwise plots. Explanatory variables appeared to be completely independent, with two exceptions. First, survey season was correlated with some of the weather variables: the juvenile season was hotter (p < 0.001, df = 204, t = -7.9) and windier (p < 0.048, df = 222, t = -2.0). The average maximum overnight temperature in the maternal season was 23.4°C, and the maximum wind speed was 24.7 km/hr. By comparison, in the juvenile season the average maximum overnight temperature 29.6°C, and the maximum wind speed was 26.8 km/hr. We chose to leave all three variables (season, temperature and wind speed) in the analysis because ‘season’ captures important life-history information that the weather variables do not. We anticipated an increase in the three bat responses in the juvenile survey period because of the increase in the number of volant individuals in the population, irrespective of weather, and this is what we found.

The second correlation relates to land use classes and habitat variables. Native pastures had lower values of habitat component 1 than other land use classes (Fig. S2a), though this component did not prove to have a very strong effect in final field models (Table 6). All land use classes differed from each another with regards to habitat component 2, with the exception of the two crop classes (cereals and canola, Fig. S2b). Once again, we chose to leave all three variables (land use, habitat component 1 and habitat component 2) in the analysis, because certain factors associated with land use class, other than habitat structure, may affect our bat responses. Crops are associated with cultivation practices such as tillage and harvesting, and both cropped fields and exotic pastures are subjected to higher inputs of pesticides, fertilisers, or herbicides which may affect bat prey. Native pastures are also less likely to have been set-stocked for long periods of time, and it is not known how this may indirectly affect bats.

**References**

1. Lumsden LF, Bennett AF, Silins JE (2002) Selection of roost sites by the lesser long-eared bat (*Nyctophilus geoffroyi*) and Gould's wattled bat (*Chalinolobus gouldii*) in south-eastern Australia. Journal of Zoology 257: 207-218.

2. Bennett A, Lumsden L, Nicholls A (1994) Tree hollows as a resource for wildlife in remnant woodlands: Spatial and temporal patterns across the northern plains of Victoria, Australia. Pacific Conservation Biology 1: 222-235.

3. Law B, Chidel M (2002) Tracks and riparian zones facilitate the use of Australian regrowth forest by insectivorous bats. Journal of Applied Ecology 39: 605-617.

4. Adams MD, Law BS, French KO (2009) Vegetation structure influences the vertical stratification of open- and edge-space aerial-foraging bats in harvested forests. Forest Ecology and Management 258: 2090-2100.

5. Gibbons P, Ayers D, Seddon J, Doyle S, Briggs S (2005) BioMetric: A terrestrial biodiversity assessment tool for the NSW Property Vegetation Plan Developer. Operational Manual. Canberra.

6. Department of Environment Water Heritage and the Arts (2008) Box gum grassy woodland project: field and training manual. Canberra.
